# Identification of RNA Transcript Makers Associated With Prognosis of Kidney Renal Clear Cell Carcinoma by a Competing Endogenous RNA Network Analysis

**DOI:** 10.3389/fgene.2020.540094

**Published:** 2020-10-15

**Authors:** Qiwei Yang, Weiwei Chu, Wei Yang, Yanqiong Cheng, Chuanmin Chu, Xiuwu Pan, Jianqing Ye, Jianwei Cao, Sishun Gan, Xingang Cui

**Affiliations:** ^1^Department of Urology, Gongli Hospital, Shanghai, China; ^2^Department of Urology, The Third Affiliated Hospital of Naval Military Medical University (Eastern Hepatobiliary Surgery Hospital), Shanghai, China; ^3^Laboratory of Nano Biomedicine and International Joint Cancer Institute, Second Military Medical University, Shanghai, China; ^4^Department of Pharmaceutical College, Naval Military Medical University, Shanghai, China

**Keywords:** kidney renal clear cell carcinoma, microRNAs, long non-coding RNAs, competing endogenous RNA network, survival prognosis

## Abstract

**Objective:**

This study aims to identify several RNA transcripts associated with the prognosis of kidney renal clear cell carcinoma (KIRC).

**Methods:**

The differentially expressed mRNAs, lncRNAs, and miRNAs (DEmRNAs, DElncRNAs, and DEmiRNAs) between KIRC cases and controls were screened based on an RNA-seq dataset from The Cancer Genome Atlas (TCGA) database. Subsequently, miRcode, miRDB, and TargetScan database were used to predict interactions between lncRNAs, miRNAs and target mRNAs. Then, a ceRNA network was built using miRNAs-mRNAs and lncRNAs-miRNAs pairs. Functional analysis of mRNAs in ceRNA was performed. Finally, the survival analysis of RNA transcripts in ceRNA network and correlation analysis for key RNA regulators were carried out.

**Results:**

There were 1527 DElncRNAs, 54 DEmiRNAs, and 2321 DEmRNAs. A ceRNA network was constructed among 81 lncRNAs, 9 miRNAs, and 197 mRNAs. Functional analysis showed that numerous mRNAs were significantly associated with regulation of cellular glucuronidation. In addition, 35 lncRNAs, 84 mRNAs and two miRNAs were significantly corelated to the survival of patients with KIRC (*P* < 0.05). Among them, miRNA-21 and miRNA-155 were negatively related to three lncRNAs (LINC00472, SLC25A5.AS1, and TCL6). Seven mRNA targets of miRNA-21 (*FASLG*, *FGF1*, *TGFBI*, *ALX1*, *SLC30A10*, *ADCY2*, and *ABAT*) and 12 mRNAs targets of miRNA-155 (*STXBP5L*, *SCG2*, *SPI1*, *C12orf40*, *TYRP1*, *CTHRC1*, *TDO2*, *PTPRQ*, *TRPM8*, *ERMP1*, *CD36*, and *ST9SIA4*) also acted as prognostic biomarkers for KIRC patients.

**Conclusion:**

We screened numerous novel prognosis-related RNA markers for KIRC patients by a ceRNA network analysis, providing deeper understandings of prognostic values of RNA transcripts for KIRC.

## Introduction

Kidney renal clear cell carcinoma (KIRC) is a major subtype of renal carcinoma and originated from renal epitheliums (Drucker, 2005). Numerous studies have estimated that the morbidity and mortality of KIRC have been gradually increasing in recent years, accounting for most cancer-related deaths (Hsieh et al., 2017; Wong et al., 2017). Clinically, surgical resection has been a powerful therapeutic option for localized KIRC. Unfortunately, approximately 30% patients with localized KIRC ultimately develop malignant metastases due to high recurrence and delayed diagnosis (Hutson and Figlin, 2007; Gupta et al., 2008). Moreover, metastatic KIRC is significantly resistant to radiotherapy and chemotherapy, resulting in a poor clinical prognosis (Zall et al., 2010; De Felice and Tombolini, 2018). Therefore, it is an urgent need to identify several novel prognostic makers, which will contribute to developing effective therapeutic strategies for improving the overall survival (OS) of patients undergoing KIRC.

Previous studies have reported that many non-coding RNA transcripts such as long non-coding RNAs (lncRNAs) and microRNAs (miRNAs) were involved in molecular pathogenesis of several types of cancer, including KIRC (Khadirnaikar et al., 2019; Ye et al., 2019). More remarkably, an increasingly large number of research groups revealed that the interplays among messenger RNAs (mRNAs), lncRNA and miRNAs played essential roles in coordinating numerous complicated molecular processes, however, the dysregulation of biological pathways will lead to pathological conditions (Karreth and Pandolfi, 2013). Specifically, lncRNAs as molecular sponges can bind to miRNAs by miRNA response elements (MREs) and consequently inhibit the expression of mRNAs. Accordingly, a competitive endogenous (ceRNA) network theory is proposed and discussed the interactions among these three types of RNA transcripts (Salmena et al., 2011). Recently, the researchers have screened many RNA transcripts targets associated with the clinical outcomes of various cancers by ceRNA regulatory network analyses. For example, Wang et al. (2019) constructed a mRNA-miRNA-lncRNA network, in which several RNA transcripts were associated with the prognosis of patients with pancreatic cancer. Similarly, Lin et al. (2018) also established a ceRNA network by a genome-wide analysis of lncRNAs, miRNAs and mRNAs, which were all related to the survival of hepatocellular carcinoma patients. Moreover, they found that this network was linked with a poor prognosis by regulating the cell cycle (Lin et al., 2018). However, few studies considered to identify prognostic RNA transcripts for KIRC patients based on a lncRNA-miRNA-mRNA ceRNA network analysis.

In this study, we conducted a differential expression analysis of three types of RNA transcripts (lncRNAs, mRNAs and miRNAs) between KIRC patients and normal controls using an RNA-seq dataset from The Cancer Genome Atlas (TCGA) database. Then, a lncRNA-miRNA-mRNA ceRNA regulatory network was established to the characterize RNA transcripts. Finally, a survival analysis was carried out to evaluate the relationships of RNA transcripts and KIRC prognosis, which will provide new insights into prognostic values of RNA transcripts for KIRC patients.

## Materials and Methods

### Acquisition of RNA-Sequencing (RNA-Seq) Data

RNA-seq raw data from patients with KIRC was downloaded from TCGA database^1^ (which consisted of three types of RNA (lncRNA, miRNA and mRNA). This dataset contained 611 samples (505 samples from KIRC patients and 106 sample from healthy individuals). Those KIRC patients with missing follow-up information were excluded from this study. Finally, RNA data and clinical data from 474 tumor samples and 106 normal samples were analyzed.

### Data Pre-processing and Differential Expression Analysis

The EdgeR package in R software has been successfully used for differential expression analysis of RNA-seq based on a negative binomial distribution model 14. The raw read counts of all samples were merged in a single read count matrix. The matrix was normalized and standardized by TMM (Trimmed Mean of M-values) method before the differential expression analysis. The TMM normalization method is implemented in the edgeR package by means of the *calcNormFactors* function. Herein, we utilized the EdgeR package to identify the differentially expressed mRNAs (DEmRNAs), lncRNAs (DElncRNAs), and miRNAs (DEmiRNAs) between KIRC tissues and normal tissues. The corresponding *P-*value of differential gene expression was calculated by Student’s *t-*test and adjusted by Benjamini and Hochberg method. The screening criteria for statistical significance were set as adjusted *P* < 0.05 and |log_2_ fold change (FC)|>2. Besides, the Volcano plots were visualized to demonstrate the distribution of differential expression of DEmRNAs, DElncRNAs and DEmiRNAs using the ggplot2 packages in R.

### Construction of lncRNA-miRNA-mRNA Network

Existing evidence has suggested that there were crosstalks among several types of RNA transcripts by MREs, including lncRNAs, miRNAs and mRNAs. Accordingly, numerous studied have demonstrated that lncRNAs act as ceRNAs sponge to bind to miRNAs through MREs, thereby regulating the expression level of mRNA (Tay et al., 2014). Therefore, a ceRNA regulatory network was established to investigate associations among DElncRNAs, DEmiRNAs, and DEmRNAs. LncRNA-miRNA-mRNA pairs should share the same miRNAs. Briefly, the miRcode^2^, a database including more than 10,000 lncRNAs and providing putative miRNA target sites on the basis of comprehensive GENCODE^3^ gene annotation, was employed to predict DElncRNA-DEmiRNA pairs (Jeggari et al., 2012). Meanwhile, the interactions between DEmiRNAs and mRNAs were assessed using experimentally validated miRTarBase^4^ and TargetScan^5^ database (Tan et al., 2009; Hsu et al., 2014). The interactions that matched with DElncRNAs and DE-mRNAs were screened. Also, miRNA-lncRNA and miRNA-mRNA interactions that did not contain the same miRNA were eliminated. Subsequently, the intersecting mRNAs between predicted mRNA targets for DEmiRNAs and DEmRNAs were obtained. Finally, we used Cytoscape software 3.5.1^6^ to construct and visualize the ceRNA network.

### Functional Enrichment Analysis of DEmRNAs in ceRNA Network

To explore the underlying biological functions of DEmRNAs from the ceRNA regulatory network, the Gene Ontology (GO) functional annotation analysis was carried out by an web-based functional annotation tool, Database for Annotation, Visualization and Integrated Discovery (DAVID)^7^ (Ashburner et al., 2000; Dennis et al., 2003). GO analysis involved in three categories of biological process (BP), cellular component (CC), and molecular function (MF). In addition, the clusterProfiler package in R was used for Kyoto Encyclopedia of Genes and Genomes (KEGG) enrichment analysis of DEmRNAs in ceRNA network (Yu et al., 2012; Kanehisa et al., 2017). The significant GO terms and KEGG pathways were selected according to the cut of *P* < 0.05.

### Survival Analyses

The correlations between DEmRNAs, DElncRNAs and DEmiRNAs of the ceRNA regulatory network and OS of patients suffering from KIRC were, respectively, assessed by Kaplan-Meier (KM) method and log-rank test using R Survival package^8^. The *P* < 0.05 was regarded as statistically significantly different. The DElncRNAs, DEmiRNAs and DEmRNAs that showed close association with 5-year survival were considered as key prognostic markers for KIRC patients.

### Correlation Analysis

Increasing studies have reported that lncRNAs-miRNAs signatures were predominately correlated with the clinical prognosis of cancer patients. Moreover, lncRNAs participate in development and progression of various diseases through negatively regulating the expression of corresponding miRNAs (Ge et al., 2015; Zhai et al., 2017; Tian et al., 2019). Here, the Pearson’s correlation analysis of prognostic factors (DElncRNAs and DEmiRNAs) was firstly conducted. Next, those DElncRNAs-DEmiRNAs pairs that exhibited negative relationships were retained for following analysis.

### Univariate and Multivariate Cox Regression Analysis

The significantly negatively regulated DElncRNAs-DEmiRNAs pairs were selected. The clinical data from 474 KIRC patients was collected and used for univariate and multivariate Cox hazards regression analysis to confirm the correlations of selected DElncRNA or DEmiRNA and the survival outcomes of patients undergoing KIRC. The correlations between the expression of key RNA transcript and different pathologic stages was evaluated. Besides, we also assessed the associations between the risk of KIRC and multiple clinical characteristics, including age, gender, pathologic stage, cancer status, neoplasm type and tumor volume. The hazard ratios (HR) with 95% confidence intervals (CI) were estimated to predict KIRC risk.

#### SiRNA Transfection

Small interfering RNAs (siRNA) targeting SLC25A5-AS1, LINC00472, and TCL6 (si-lnc#1 and si-lnc#2 respectively) and control siRNAs were all subscribed from Sangong Biological corporation (Songjiang, Shanghai, China). The cell transfection was carried out by the utilizing of Lipofectamine 3000 reagent kits (Invitrogen, Waltham, MA, United States) according to the protocols in the kits.

#### Quantitative Real Time-PCR

KIRC cells transfected with siRNAs or corresponding vectors were gathering 48 h later after incubation. Subsequently, the total RNAs were extracted from cultured cells utilizing TRIzol reagent (TaKaRa Bio Inc., Otsu Prince Hotel, Japan) for Real Time-PCR analyses. Reverse transcription of converting purified RNA to complementary DNA (cDNA) was carried out by applying the BeyoRT II cDNA synthesis kits (Beyotime Biotechnology, shanghai, China). The cDNAs were confirmed to qPCR experiments with utilizing the SYBR Green qRCR kits (TaKaRa Bio Inc., Otsu Prince Hotel, Japan) according to the manufacturer’s recommendations.

#### Cell Proliferation Assays

The cell growth was detected in accordance with CCK-8 kit (DOJINDO, kyushu, Japan). Briefly, 786O cells were placed into ninety-six well plates (2000 cells per well). At assigned timepoint, CCK8 reagents were added into the wells (10 μl per well). After incubated at 37°C in 5c/o CO_2_ for 3.5 h, the absorbance was detected at 450 nm on a microplate spectrophotometer.

## Results

### Identification of DElncRNAs, DEmiRNAs, and DEmRNAs,

**FIGURE 1:**
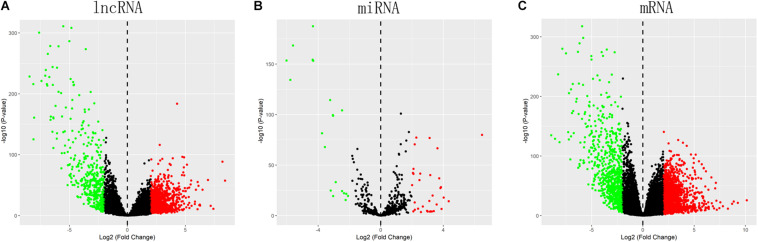
Volcano plots show differential expression of RNAs between KIRC group and control group. **(A)** DElncRNAs; **(B)** DEmiRNAs; **(C)** DEmRNAs. The red color shows the up-regulated lncRNAs, miRNAs, and mRNAs while the green color represents down-regulated lncRNAs, miRNAs, and mRNAs. KIRC: kidney renal clear cell carcinoma. DElncRNAs: differentially expressed lncRNAs; DEmiRNAs: differentially expressed miRNAs; DEmRNAs: differentially expressed mRNAs.

Totally, 1527 DElncRNAs (1068 up-regulated lncRNAs and 459 down-regulated lncRNAs; Table 1), 54 DEmiRNAs (33 up-regulated miRNAs and 21 down-regulated miRNAs; Table 2), and 2321 DEmRNAs (1557 up-regulated mRNAs and 764 down-regulated mRNAs; Table 3) between KIRC group and control group were extracted according to pre-determined screening criteria. Volcano plots for DElncRNAs, DEmiRNAs, and DEmRNAs were visualized and showed their distributions (Figure 1). All of DElncRNAs, DEmiRNAs, and DEmRNAs were list in Supplementary Table S1.

**TABLE 1:** Top 30 DElncRNAs between tumor and normal tissues in KIRC.

Symbol	LogFC	*P*-value	FDR	Type
AC019080.1	−3.848634083	0	0	Down
AL031726.1	−5.575757888	4.30 e–313	0.00E + 00	Down
AP005432.2	−6.750172893	0	0	Down
LINC01762	−4.852411641	6.62E–306	1.49E–302	Down
AC090709.1	−7.637409071	7.30E–301	1.31E–297	Down
AP000696.1	−5.981997305	5.14E–282	7.71E–279	Down
AC009035.1	−6.685506981	2.87E–277	3.69E–274	Down
AC068631.1	−5.002329371	9.57E–276	1.08E–272	Down
AC023421.1	−6.875173385	1.50E–263	1.50E–260	Down
LINC01378	−6.482416569	3.97E–244	3.58E–241	Down
AC007993.2	−6.09790285	2.54E–241	2.08E–238	Down
AC073172.1	−7.06091573	4.42E–236	3.32E–233	Down
AC079310.1	−8.461310238	6.09E–230	4.22E–227	Down
AL139280.1	−6.76699975	6.26E–229	4.03E–226	Down
AC105384.1	−4.946354427	5.55E–228	3.33E–225	Down
LINC01571	−7.095216192	1.30E–226	7.30E–224	Down
LINC02410	−4.722320287	3.38E–219	1.79E–216	Down
AC124017.1	−7.417576168	1.56E–218	7.81E–216	Down
AC006441.4	−4.644381271	2.25E–216	1.07E–213	Down
LINC01020	−6.476526915	4.19E–216	1.89E–213	Down
AC092078.2	−6.969671309	1.96E–215	8.38E–213	Down
LINC02437	−8.122528406	2.94E–214	1.20E–211	Down
WSPAR	−4.675867025	4.09E–213	1.60E–210	Down
AC016526.1	−6.93485957	1.31E–212	4.90E–210	Down
AC008264.2	−3.212867326	1.98E–205	7.15E–203	Down
AC092078.1	−5.752136263	1.08E–202	3.73E–200	Down
AC005165.1	−4.175726425	1.77E–201	5.90E–199	Down
AC023154.1	−5.939176939	9.41E–198	3.03E–195	Down
AC079340.2	−4.782821842	1.20E–191	3.74E–189	Down
AL359317.2	−2.883987888	3.20E–186	9.61E–184	Down

**TABLE 2:** Top 30 DEmiRNAs between tumor and normal tissues in KIRC.

Symbol	LogFC	*P*-value	FDR	Type
hsa-mir-508	−4.330852316	3.90E–188	1.91E–185	Down
hsa-mir-506	−5.604961065	4.48E–169	1.10E–166	Down
hsa-mir-514a-1	−4.349319617	5.14E–155	8.39E–153	Down
hsa-mir-514a-3	−4.352581963	9.52E–155	1.17E–152	Down
hsa-mir-514b	−6.016927898	3.42E–154	3.35E–152	Down
hsa-mir-514a-2	−4.314090398	5.99E–154	4.90E–152	Down
hsa-mir-934	−5.773695239	4.56E–135	3.19E–133	Down
hsa-mir-509-3	−3.232081398	4.13E–115	2.53E–113	Down
hsa-mir-362	−2.472895463	6.26E–105	3.41E–103	Down
hsa-mir-509-1	−3.060183569	3.01E–100	1.34E–98	Down
hsa-mir-509-2	−3.038963476	1.67E–99	6.81E–98	Down
hsa-mir-129-1	−3.752751281	3.62E–82	1.27E–80	Down
hsa-mir-122	6.469661663	1.23E–80	4.01E–79	Up
hsa-mir-21	2.273110818	6.81E–78	2.09E–76	Up
hsa-mir-210	3.123123884	1.38E–77	3.97E–76	Up
hsa-mir-584	2.168406613	3.04E–71	7.45E–70	Up
hsa-mir-129-2	−3.566439193	1.40E–68	3.27E–67	Down
hsa-mir-155	3.621988091	2.47E–67	5.49E–66	Up
hsa-mir-4772	2.095164137	5.07E–47	8.02E–46	Up
hsa-mir-452	2.055067567	5.35E–43	7.71E–42	Up
hsa-mir-224	2.523169474	3.06E–42	4.17E–41	Up
hsa-mir-142	2.091904473	5.48E–42	7.26E–41	Up
hsa-mir-592	3.162999771	7.92E–41	9.47E–40	Up
hsa-mir-885	3.734178616	1.22E–37	1.36E–36	Up
hsa-mir-3941	2.470576003	7.93E–36	7.62E–35	Up
hsa-mir-200c	−2.875603571	7.18E–34	6.63E–33	Down
hsa-mir-6509	2.003843743	7.59E–31	6.30E–30	Up
hsa-mir-4773-1	3.849423442	6.36E–29	4.94E–28	Up
hsa-mir-4773-2	3.81455964	6.62E–28	4.91E–27	Up
hsa-mir-216b	−3.199337565	1.49E–25	1.06E–24	Down

**TABLE 3:** Top 30 DEmRNAs between tumor and normal tissues in KIRC.

Symbol	LogFC	*P*-value	FDR	Type
SIM2	−4.499143313	0	0	Down
MFSD4A	−5.237800114	0	0	Down
ACPP	−5.56624401	0	0	Down
LINC00675	−7.078400696	0	0	Down
GPC5	−5.826905966	5.57E–299	1.68E–295	Down
KCNJ10	−6.175437553	7.17E–290	1.85E–286	Down
ELF5	−7.85373104	7.41E–281	1.67E–277	Down
ADGRF3	−3.527399864	2.25E–279	4.53E–276	Down
DDN	−6.340138124	1.61E–275	2.92E–272	Down
MYLK3	−4.024392554	3.88E–275	6.37E–272	Down
ATP1A1	−2.749724478	5.48E–275	8.26E–272	Down
CALB1	−7.504555812	5.02E–273	6.98E–270	Down
HSPA2	−3.886135943	1.02E–268	1.31E–265	Down
IRX2	−5.022270965	1.37E–268	1.65E–265	Down
ENPP6	−5.018567582	1.26E–262	1.43E–259	Down
SLC12A1	−8.265248669	8.25E–238	8.78E–235	Down
MTURN	−3.02727238	2.59E–237	2.60E–234	Down
SLC4A11	−4.438131102	5.08E–236	4.83E–233	Down
TRPV6	−4.865208043	2.55E–230	2.19E–227	Down
FGF1	−4.20176033	4.96E–228	4.07E–225	Down
GSTM3	−3.298323834	9.20E–225	7.23E–222	Down
HS6ST2	−6.247025775	2.81E–222	2.11E–219	Down
DUSP9	−6.832840389	1.10E–221	7.95E–219	Down
CLDN16	−5.840504447	3.42E–220	2.38E–217	Down
UNCX	−7.014507826	1.24E–212	8.34E–210	Down
C14orf37	−3.54316015	1.18E–206	7.60E–204	Down
ATP12A	−7.212349725	3.24E–206	2.02E–203	Down
WNK4	−4.070609116	2.13E–203	1.29E–200	Down
EGF	−5.420543201	1.52E–201	8.86E–199	Down
HS6ST1	−2.144958399	2.42E–200	1.32E–197	Down

### Construction of ceRNA Network

**FIGURE 2:**
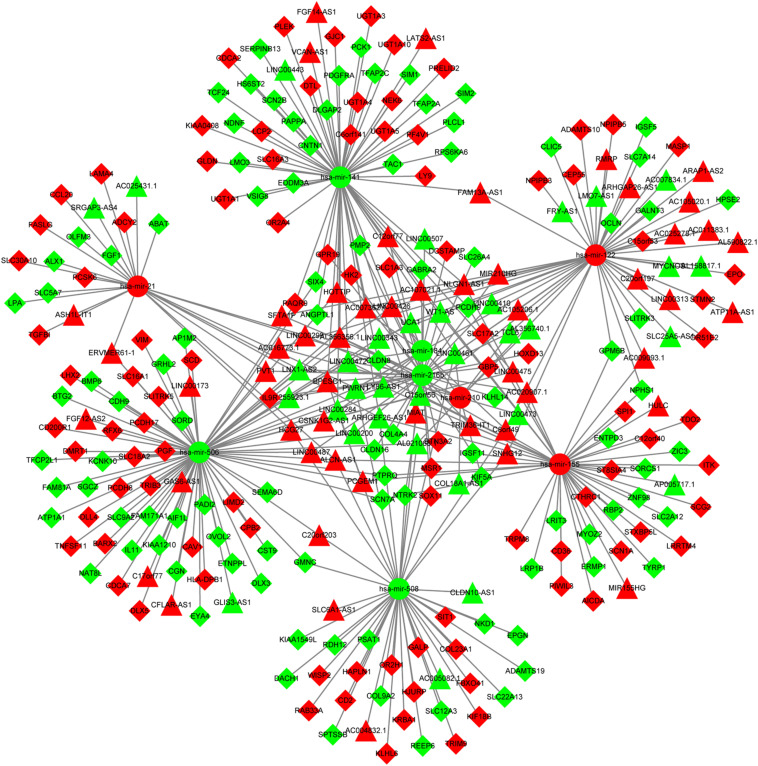
The ceRNA network of lncRNA-miRNA-mRNA. The circles represent miRNAs. The triangles show lncRNAs. The rhombuses represent mRNAs. The red nodes show up-regulated expression while the green nodes represent down-regulated expression. ceRNA, competing endogenous RNA; lncRNA, long non-coding RNA; miRNA, microRNA.

The predictive analysis for interactions between DElncRNAs and DEmiRNAs revealed that there were 174 DElncRNAs-DEmiRNAs pairs, including 81 DElncRNAs and 9 DEmiRNAs. Furthermore, two databases (miRTarBase and TargetScan) were utilized to predict the mRNA targets of 9 DEmiRNAs and a total of 197 DEmRNAs were intersected with predicted mRNAs. Afterward, 212 pairs of DEmiRNAs and DEmRNAs were determined, including 197 DEmRNAs and 9 DEmiRNAs. Ultimately, the DElncRNAs-DEmiRNAs-DEmRNAs ceRNA network was constructed using Cytoscape 3.5.1, which contained 81 DElncRNAs, 9 DEmiRNAs and 197 DEmRNAs (Supplementary Table S2 and Figure 2).

### Functional Analyses

The GO and KEGG functional analysis were carried out to understand potential roles of DEmRNAs on KIRC. We found that these mRNAs were involved in 64 GO-BP terms, 17 GO-CC terms and 19 GO-MF terms. Specifically, the top three GO-BP terms were xenobiotic glucuronidation, flavonoid biosynthetic process and negative regulation of cellular glucuronidation (Supplementary Table S3). Meanwhile, the overwhelming majority of mRNAs were predominately correlated with three GO-CC terms, including integral component of membrane, proteinaceous extracellular matrix and extracellular space (Supplementary Table S3). For GO-MF analysis, the mRNAs played essential roles in multiple significantly enriched terms, such as glucuronosyltransferase activity and retinoic acid binding (Supplementary Table S3). In addition, KEGG enrichment analysis suggested that the mRNAs were markedly enriched in 16 KEGG pathways, including pentose and glucuronate interconversions, ascorbate and aldarate metabolism and retinol metabolism pathway (Supplementary Table S3).

### Survival Analysis

**FIGURE 3:**
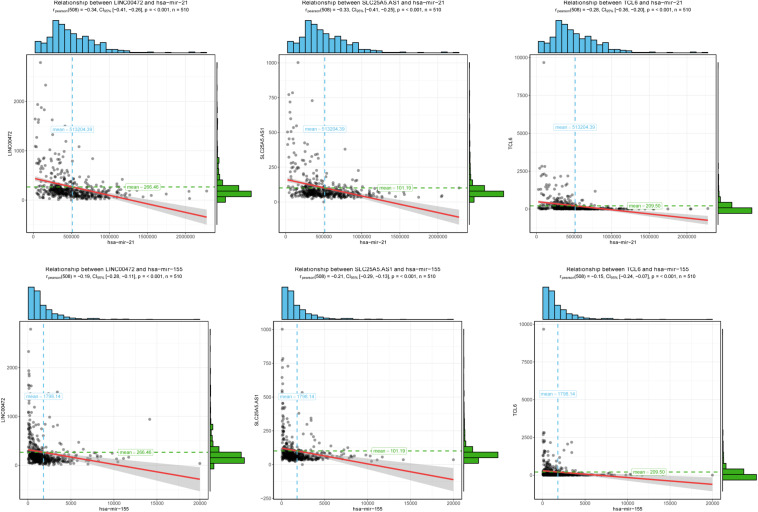
The correlation analysis of two miRNAs and corresponding negatively related lncRNAs. Two miRNAs (miRNA-21 and miRNA-155) negatively interacted with three lncRNAs (LINC00472, SLC25A5.AS1, and TCL6). The horizontal axis represents miRNAs and vertical axis represents lncRNAs.

**FIGURE 4:**
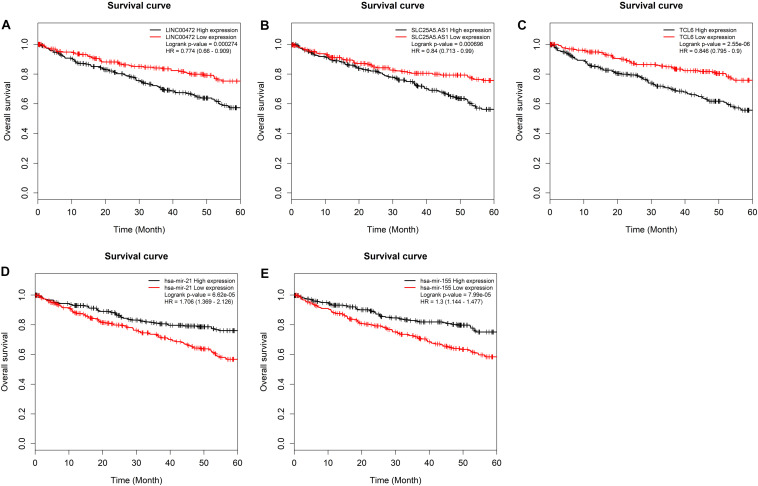
Kaplan–Meier survival curves for two miRNAs and three lncRNAs. **(A)** LINC00472, **(B)** SLC25A5.AS1, **(C)** TCL6, **(D)** miRNA-21, and **(E)** miRNA-155. The horizontal axis presents the survival time while the vertical axis presents the survival rate. The green curve represents the low expression group while black curve shows high expression group. MiRNAs, microRNAs; lncRNAs, long non-coding RNAs.

**FIGURE 5:**
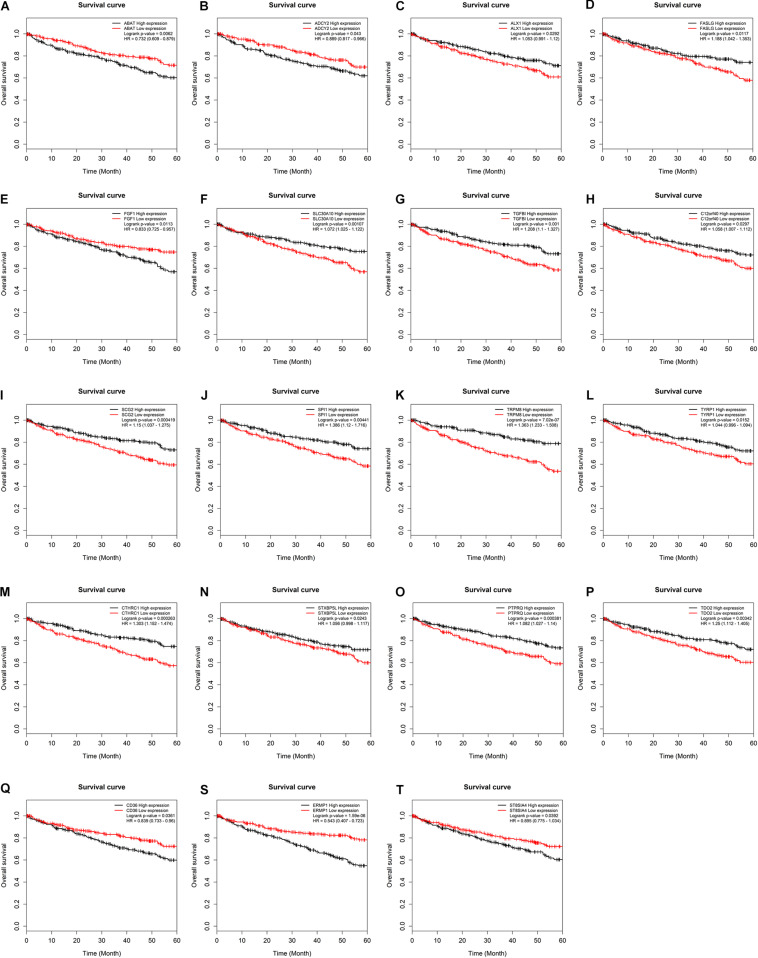
Kaplan–Meier survival curves for key mRNAs. **(A)** ABAT, **(B)** ADCY2, **(C)** ALX1, **(D)** FASLG, **(E)** FGF1, **(F)** SLC30A10, **(G)** TGFBI, **(H)** C12orf40, **(I)** SCG2, **(J)** SPI1, **(K)** TRPM8, **(L)** TYRP1, **(M)** CTHRC1, **(N)** STXBP5L, **(O)** PTPRQ, **(P)** TDO2, **(Q)** CD36, **(S)** ERMP1, **(T)** ST8SIA4. The horizontal axis presents the survival time while the vertical axis presents the survival rate. The green curve represents the low expression group while black curve shows high expression group.

To further assess the potential prognostic values of lncRNAs, miRNAs, and mRNAs in ceRNA, the corresponding KM survival analyses were conducted. The results indicated that 35 lncRNAs, 84 mRNAs and two miRNAs (up-regulated miRNA-21 and miRNA-155) were dramatically related to 5-year survival of patients undergoing KIRC (*P* < 0.05). More notably, lncRNAs participate in molecular mechanisms of cancerogenesis through sponging miRNAs, in which lncRNAs are generally negatively associated with miRNAs. Herein, we performed a Pearson’s correlation coefficient analysis between prognosis-related lncRNAs and miRNAs. The results revealed that there were significantly negative correlations between miRNA-21 and 14 lncRNAs. Moreover, miRNA-21-LINC00472 (*P* = −0.337), miRNA-21-SLC25A5.AS1 (*P* = −0.330) and miRNA-21-TCL6 (*P* = −0.284) were top three pairs with the strongest relationships (Supplementary Table S4 and Figure 3). For miRNA-155, we noted that it was inversely related to 10 lncRNAs (Supplementary Table S4). Interestingly, there were also the most dramatical interactions between miRNA-155 and LINC00472, SLC25A5.AS1, and TCL6 (miRNA-155-LINC00472, *P* = −0.212; miRNA-21-SLC25A5.AS1, *P* = −0.193; and miRNA-21-TCL6, *P* = −0.154; Supplementary Table S4 and Figure 3). Moreover, we noted that higher expression of miRNA-21 and miRNA-155 exhibited better clinical outcomes while decreased levels of three lncRNAS (LINC00472, SLC25A5.AS1, and TCL6) were strongly associated with good prognosis by the survival analysis, which implied that these RNA transcripts may act as pivotal risk factors in KIRC progression (Figure 4). In addition, there were close relationships between miRNA-21 and seven prognosis-related mRNAs in ceRNA regulatory network, including adenylate cyclase 2 (*ADCY2*), down-regulated 4-aminobutyrate aminotransferase (*ABAT*), fibroblast growth factor 1 (*FGF1*), up-regulated fas ligand (*FASLG*), transforming growth factor beta induced (*TGFBI*), ALX homeobox 1 (*ALX1*), and solute carrier family 30 member 10 (*SLC30A10*). Moreover, we discovered that increased expression of *FASLG*, *FGF1*, *TGFBI*, *ALX1*, and *SLC30A10* showed favorable clinical survival but raised levels of *ADCY2* and *ABAT* indicated worse outcomes (Figures 5A–G). Similarly, miRNA-155 strongly interacted with 12 prognosis-associated mRNAs according to ceRNA analysis, including syntaxin binding protein 5 like (*STXBP5L*), secretogranin II (*SCG2*), endoplasmic reticulum metallopeptidase 1 (*ERMP1*), Spi-1 proto-oncogene (*SPI1*), chromosome 12 open reading frame 40 (*C12orf40*), tyrosinase related protein 1 (*TYRP1*), collagen triple helix repeat containing 1 (*CTHRC1*), tryptophan 2,3-dioxygenase (*TDO2*), CD36 molecule (*CD36*; up-regulation), ST8 alpha-N-acetyl-neuraminide alpha-2,8-sialyltransferase 4 (*ST8SIA4*), protein tyrosine phosphatase receptor type Q (*PTPRQ*), and transient receptor potential cation channel subfamily M member 8 (*TRPM8*). Higher expressions of nine mRNAs (*STXBP5L*, *SCG2*, *SPI1*, *C12orf40*, *TYRP1*, *CTHRC1*, *TDO2*, *PTPRQ*, and *TRPM8*) were correlated with better survival (Figures 5H–P). However, elevated expression levels of *ERMP1*, *CD36* and *ST8SIA4* were linked with poor prognosis (Figures 5Q–T).

### Risk Evaluation of the Key lncRNA and miRNA With Overall Survival of KIRC Patients

**FIGURE 6:**
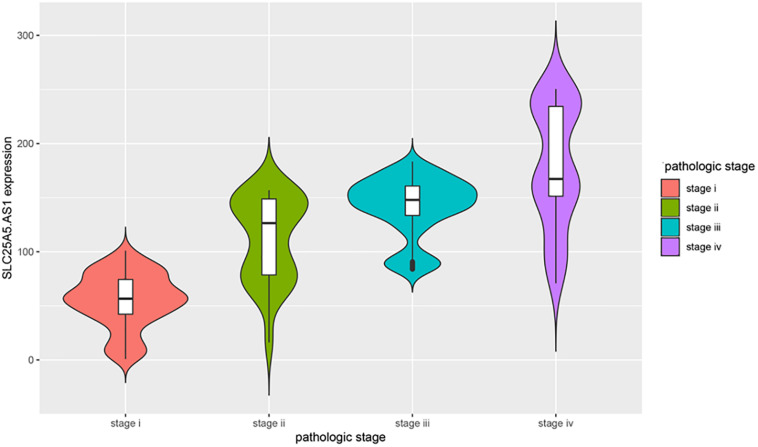
The violin plot of the relationships between SLC25A5.AS1 expression and pathological stages. The correlations between four pathological stages and the expression level of SLC25A5.AS1 were evaluated. The horizontal axis presents the different pathological stage while the vertical axis presents the expression level of SLC25A5.AS1.

Many previous studies suggested that LINC00472 and miRNA-21 served as significant predictors for the survival of KIRC patients (Zaman et al., 2012; Yang et al., 2018; Yu et al., 2018). We assessed the associations between key RNA transcripts (SLC25A5-AS1 and miRNA-21) and the clinical survival of patients with KIRC. The corresponding *P*-values with 95% CI were calculated to predict patients’ survival risk. We found that SLC25A5-AS1 and miRNA-21 were strongly related to the risk of KIRC occurrence according to the univariate Cox analysis (SLC25A5-AS1, HR = 0.610, 95% CI: 0.438–0.848, *P* = 0.003; miRNA-21, HR = 1.908, 95% CI: 1.37–2.657, *P* < 0.001; Table 4). Furthermore, the multivariate Cox analysis indicated that SLC25A5-AS1 was the independent prognostic factor of KIRC (HR = 0.836, 95% CI: 0.587–1.190, *P* = 0.032; Table 4). Besides, the relationships between clinicopathological features and KIRC risk were also evaluated. Our findings showed that age, pathological stage and cancer status were dramatically associated with the risk of KIRC patients (Table 4). Afterward, the correlations between SLC25A5-AS1 and the clinical features of patients with KIRC were also evaluated. The results showed that SLC25A5-AS1 was related to clinical stage, pathological stage and tumor volume (Table 5). Accordingly, we observed that there was the highest expression of SLC25A5-AS1 in KIRC patients under pathological stage IV, suggesting that SLC25A5-AS1 may be a key target for predicting KIRC progression (Figure 6).

**TABLE 4:** Univariate and multivariate analyses of clinicopathological characteristics, SLC25A5-AS1 and miRNA-21 with overall survival in TCGA KIRC cohort.

Indicators	Univariate analysis		Multivariate analysis	
		
TCGA KIRC set (*n* = 474)	HR (95% CI)	*P-*value	HR (95% CI)	*P-*value
Age	0.527(0.378−0.735)	<0.001	0.566(0.403−0.795)	0.001
Gender	1.08(0.771−1.511)	0.655		
Pathologic_stage	3.718(2.635−5.248)	<0.001	2.488(1.155−5.358)	0.0199
Pathologic_T	2.982(2.145−4.145)	<0.001	0.684(0.356−1.315)	0.255
Pathologic_N	0.823(0.596−1.136)	0.237		
Pathologic_M	3.749(2.688−5.228)	<0.001	1.249(0.823−1.895)	0.2954
Cancer_status	5.688(4.037−8.013)	<0.001	4.021(2.635−6.136)	<0.001
Neoplasm_type	2.632(1.808−3.832)	<0.001	1.473(0.958−2.263)	0.0774
Tumor_volume	0.434(0.308−0.613)	<0.001	1.311(0.852−2.018)	0.2176
MiRNA-21	1.908(1.37−2.657)	<0.001	1.42(0.985−2.048)	0.0602
SLC25A5-AS1	0.61(0.438−0.848)	0.003	0.836(0.587−1.19)	0.03206

**HR, hazard ratio. CI, confidence interval.**

**TABLE 5:** The relationship between CRHBP and clinical features with KIRC patients in TCGA.

	Cases (N)	Low (N)	High (N)	*P-*value
Sample	474	237	237	
Age				0.7828
≤60 year	232	118	114	
>60 year	242	119	123	
Gender	0			0.1451
Male	314	149	165	
Female	160	88	72	
Clinical stage	0			0.0019*
I	233	125	108	
II	48	37	11	
III	115	42	73	
IV	78	33	45	
Pathologic T	0			0.0015*
T1	238	130	108	
T2	59	30	49	
T3	167	52	115	
T4	10	5	5	
Pathologic N	0			0.9266
N0	218	108	110	
N1	256	129	127	
Pathologic M	0			0.2585
M0	375	193	182	
M1	99	44	55	
Cancer status	0			0.1294
Yes	336	176	160	
No	138	61	77	
Histologic grade	0			0.1308
G1	12	5	7	
G2	199	115	84	
G3	188	92	96	
G4	75	25	50	
Tumor volume	0			0.0431*
≤5.5 cm (median)	227	102	125	
>5.5 cm	247	135	112	

* Represents P-value < 0.05.

#### The Expression of SLC25A5-AS1, LINC00472, and TCL6 in KIRC Specimens

**FIGURE 7:**
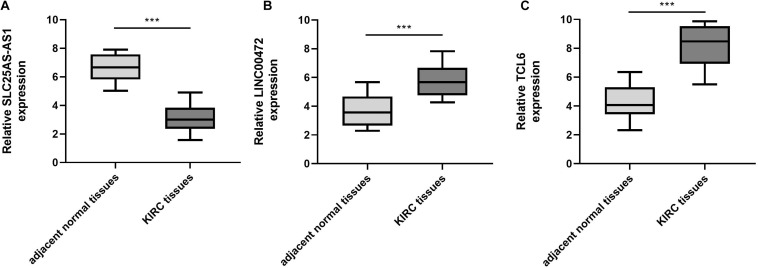
SLC25A5-AS1 was under-expressed, LINC00472 and TCL6 was highly expressed, in KIRC tissues in comparison to adjacent normal tissues. **(A–C)** qRT-PCR was performed for the examination of SLC25A5-AS1, LINC00472 and TCL6 levels in KIRC tissues and adjacent normal tissues. *p < 0.05, **p < 0.01, ***p < 0.001. KIRC, kidney renal clear cell carcinoma.

To verify the bioinformatics analysis, we performed qRT-PCR assays to evaluate the SLC25A5-AS1, LINC00472, and TCL6 levels in KIRC samples and adjacent normal tissues. The results illuminated that the SLC25A5-AS1 levels were down-regulated in KIRC samples in comparison to the adjacent normal tissues (Figure 7A). Nevertheless, LINC00472 and TCL6 were up-regulated in KIRC samples compared with adjacent normal tissues (Figures 7B,C).

#### Knocking Out SLC25A5-AS1, LINC00472, and TCL6 Will Inhibit or Promote the Proliferation of KIRC Cells

**FIGURE 8:**
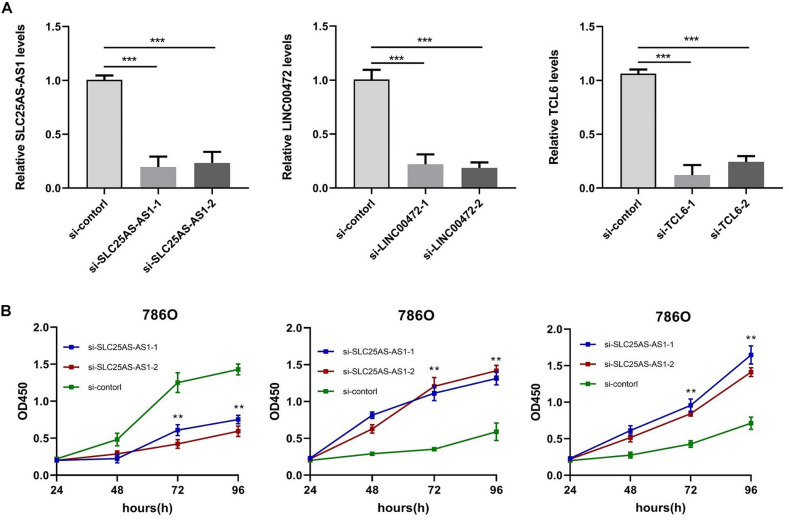
SLC25A5-AS1 promoted, LINC00472 and TCL6 inhibited, proliferation of 786O cells. **(A)** SLC25A5-AS1, LINC00472, and TCL6 levels in 786O cells were measured by qRT-PCR analyses. **(B)** CCK8 assays detected the cellular growth of 786O cells when SLC25A5-AS1, LINC00472, and TCL6 were knocked out. *p < 0.05, **p < 0.01, ***p < 0.001.

To demonstrate the effect of SLC25A5-AS1, LINC00472, and TCL6 on cellular growth of KIRC cells, we carried out loss of function studies using SLC25A5-AS1, LINC00472, and TCL6 siRNAs. Real Time-PCR analysis showed that SLC25A5-AS1 siRNAs, LINC00472 siRNAs, and TCL6 siRNAs, which constructed si-lnc#1 and si-lnc#2 respectively, successfully knocked out the expression of SLC25A5-AS1, LINC00472, and TCL6 in KIRC cells (Figure 8A). Subsequently, the results from CCK-8 experiments uncovered that the proliferation of KIRC cells were obviously suppressed by silencing SLC25A5-AS1 expression. Nonetheless, the growth of KIRC cells were notably promoted by depressing LINC00472 and TCL6 expression (Figure 8B).

## Discussion

In the present study, we performed a comprehensive bioinformatics analysis using RNA-seq data from TCGA database to identify key RNA transcript signatures associated with the clinical survival of KIRC patients. Totally, 1527 DElncRNAs, 54 DEmiRNAs, and 2321 DEmRNAs were screened between KIRC group and control group. Subsequently, a lncRNA-mediated ceRNA network was built among 81 DElncRNAs, 9 DEmiRNAs and 197 DEmRNAs. Moreover, 35 lncRNAs, 84 mRNAs, and two miRNAs (up-regulated miRNA-21 and miRNA-155) were significantly associated with OS of patients with KIRC. Moreover, there were the strongest negative interactions between miRNA-21/miRNA-155 and three prognosis-related lncRNAs (LINC00472, SLC25A5.AS1, and TCL6). Accordingly, the Cox regression analysis also revealed that SLC25A5-AS1 and miRNA-21 were dramatically linked with KIRC risk.

SLC25A5-AS1 is an anti-sense lncRNA and located in X chromosome q24. Our result suggested SLC25A5-AS1 was down-regulated in KIRC patients compared to those healthy individuals. Moreover, SLC25A5-AS1 was closely related to the risk of KIRC Consistent with this, Yang et al. (2018) previously also constructed a ceRNA network based on a TCGA dataset and evaluated prognostic values of significant lncRNAs. They emphasized that SLC25A5-AS1 was under-expressed in KIRC tissues in comparison to adjacent normal tissues and associated with OS of KIRC patients. Notably, their finding showed an increased expression SLC25A5-AS1 was related to a favorable prognosis. However, our survival analysis indicated that a lower expression of SLC25A5-AS1 exhibited a better clinical outcome for patients suffering from KIRC. Possible explanations were the differences from sample size and the duration of follow-up. Interestingly, we also noted that SLC25A5-AS1 expression was positively correlated with pathogenic stage. There was the highest level of SLC25A5-AS1 in KIRC patients under pathogenic IV. Additionally, Li et al. (2019) pointed out SLC25A5-AS1 sponging miR-19a-3p restrained gastric cancer cell growth but increased apoptosis through PTEN/PI3K/AKT signaling pathway. We noted that SLC25A5-AS1 negatively interacted with miRNA-21. Differential expression analysis suggested that there was a higher level of miRNA-21 in KIRC samples than normal controls. Moreover, elevated expression of miRNA-21 was correlated with good survival outcomes. A growing body of research has demonstrated that miRNA-21 acted as a key predictor for KIRC. For example, Szabó et al. (2016) revealed that miRNA-21 was over-expressed in KIRC and strongly linked with metastasis and survival of KIRC patients. Later on, Xie et al. (2018) argued that a four-miRNA (miRNA-21-5p, miRNA-9-5p, miR-149-5p, and miRNA-30b-5p) axis could predict the clinical outcomes of patients undergoing KIRC. These studies provided direct evidence for our conclusion that miRNA-21 was a promising prognostic factor for KIRC. We also found that miRNA-21 closely interacted with seven mRNAs (*ADCY2*, *ABAT*, *FGF1*, *FASLG*, *TGFBI*, *ALX1*, and *SLC30A10*) in ceRNA regulatory network. Accumulating evidence demonstrated that *FASLG*, a member of the tumor necrosis factor superfamily, participated in the pathogenetic mechanisms of KIRC (Elsässer-Beile et al., 2003; Donskov et al., 2004; Xu T.R. et al., 2019).

Sejima et al. (2012) highlighted that there was a remarkable worse survival for renal cell carcinomas (RCC) patients with *FASLG* mRNA-positive expression after radical nephrectomy in comparison to those with *FASLG* mRNA-negative expression, which was similar to our finding that *FASLG* expression was increased in KIRC patients and decreased level of *FASLG* exhibited good clinical prognosis. A*BAT* is central to the catabolism of gamma-aminobutyric acid, an inhibitory neurotransmitter in the mammalian central nervous system (Owens and Kriegstein, 2002). Chen et al. (2018) firstly discovered *ABAT* was dysregulated in patients with RCC compared with healthy controls and involved in the propanoate metabolism pathway by analyzing two microarray datasets (GSE53757 and GSE40435). Herein, our analysis showed that *ABAT* was down-regulated and its under-expression of *ABAT* was related to favorable prognosis of KIRC patients. *TGFBI* is located on chromosome 5q31.1, which played pivotal roles in pathologic responses of various cancers including RCC (Matsuda et al., 2008; Boguslawska et al., 2016; Pan et al., 2018). We found that higher *TGFBI* expression showed a better clinical prognosis for patients with KIRC. Moreover, elevated levels of *FGF1*, *ALX1*, and *SLC30A10* were correlated with oncogenic outcomes while decreased level of *ADCY2* was linked with good outcomes for KIRC patients. However, few groups considered the potential associations between these gene makers and survival prognosis of patients suffering from KIRC patients.

Our results also revealed that other two key lncRNAs (up-regulated LINC00472 and TCL6) may be underlying prognostic makers for the KIRC patient survival. The KIRC patients with lower expression of LINC00472 and TCL6 had better prognosis. Similarly, Wang et al. also argued that LINC00472 and TCL6 acted as independent prognostic factors for predicting clinical outcomes of KIRC patients (Wang et al., 2018). Interestingly, these two lncRNAs were strongly associated with miRNA-21 and miRNA-155. Previous studies suggested that miRNA-155 was implicated with pathogenesis of renal carcinogenesis (Klimczak et al., 2017; Shiomi et al., 2019). Chen et al. conducted an integrative bioinformatics analysis based on a Gene Expression Omnibus dataset and indicated that miRNA-15 was involved in initiation and development of KIRC (Chen et al., 2016). Our analysis showed that the over-expression of miRNA-15 was significantly linked with good prognosis. More remarkably, there was close relationships between miRNA-15 and 12 mRNAs (*STXBP5L*, *SCG2*, *SPI1*, *C12orf40*, *TYRP1*, *CTHRC1*, *TDO2*, *PTPRQ*, *TRPM8*, *ERMP1*, *CD36*, and *ST9SIA4*). Interestingly, increased expressions of *STXBP5L*, *SCG2*, *SPI1*, *C12orf40*, *TYRP1*, *CTHRC1*, *TDO2*, *PTPRQ*, and *TRPM8* were correlated with good survival outcomes but elevated expressions of *ERMP1*, *CD36*, and *ST9SIA4* were associated with poor prognosis. Xu et al. previously reported that *CD36* mRNA expression was elevated and the high expression of *CD36* represented poor prognosis in KIRC patients, which was consistent with our finding (Xu W.H. et al., 2019). However, the relevant investigations regarding the relationships between survival outcomes of KIRC patients and other gene prognostic makers are lacking. Therefore, additional analyses still need to be performed to verify our conclusion.

Although our study has identified several RNA makers for the prognostic prediction of KIRC patients, there are still some limitations. Firstly, a comprehensive bioinformatics analysis with a larger sample size is still required to carried out to validate our results. Secondly, the relevant experimental evidence was also provided for our findings. Thirdly, the more detailed clinical information needed to be integrated into a large-scale prognosis analysis in the following research.

## Conclusion

In summary, we identified 1527 DElncRNAs, 54 DEmiRNAs, and 2321 DEmRNAs between KIRC group and control group. Furthermore, a lncRNA-mediated ceRNA network was constructed. Besides, two miRNAs (miRNA-21 and miRNA-155) and corresponding three negatively correlated lncRNAs (LINC00472, **SLC25A5.AS1,** and TCL6) were associated with OS of patients with KIRC. Finally, seven mRNA targets of miRNA-21 and 12 mRNAs targeted by miRNA-155 were also pivotal prognostic makers for KIRC patients. Collectively, this study revealed several novel prognosis-related RNA makers for KIRC based on a ceRNA regulatory network, which provided deeper insights into developing promising therapeutic strategies for KIRC treatment.

## Data Availability Statement

The datasets generated for this study can be found in the TCGA database (https://cancergenome.nih.gov/), the miRcode (http://www.mircode.org/), the miRcode (http://www.mircode.org/), comprehensive GENCODE (http://www.gencodegenes.org/), experimentally validated miRTarBase (http://mirtarbase.mbc.nctu.edu.tw), TargetScan (http://www.targetscan.org/) database.

## Author Contributions

QY and WC designed the study strategy and prepared the draft of the manuscript. WY and YC acquired the data and interpreted the data. JC and JY analyzed the data and prepared the figures and tables. XP and CC managed the data and collected the references. SG and XC revised the draft of the manuscript. All authors have agreed to the final content.

## Conflict of Interest

The authors declare that the research was conducted in the absence of any commercial or financial relationships that could be construed as a potential conflict of interest.

## Footnotes

1 https://cancergenome.nih.gov/

2 http://www.mircode.org/

3 http://www.gencodegenes.org/

4 http://mirtarbase.mbc.nctu.edu.tw

5 http://www.targetscan.org/

6 https://cytoscape.org/

7 https://david.ncifcrf.gov/

8 http://CRAN.R-project.org/package=survival/

## Supplementary Material

Supplementary Table 1 All of DELncRNAs, DEmiRNA, DEmRNAs between tumor and normal tissue in KIRC.

Supplementary Table 2 Interactions of the ceRNA network in KIRC.

Supplementary Table 3 The functional enrichment analysis of differentially expressed mRNAs in ceRNA network.

Supplementary Table 4 The relationships of miRNA-21/miRNA-155 and corresponding lncRNAs.
