# Supplementary material for: Identification of RNA Transcript Makers Associated With Prognosis of Kidney Renal Clear Cell Carcinoma by a Competing Endogenous RNA Network Analysis
Source: Front Genet. 2020 Oct 15;11:540094. doi: 10.3389/fgene.2020.540094 (PMC7593646; doi:10.3389/fgene.2020.540094)
Supplement: Supplementary Table 3 — The functional enrichment analysis of differentially expressed mRNAs in ceRNA network. [file Table_1.docx]

**Table S3 The functional enrichment analysis of differentially expressed mRNAs in ceRNA network.**

| **Category** | **Term** | **Count** | ***P* value ^**^** | **Genes** |
| --- | --- | --- | --- | --- |
| GO-BP^*^ | xenobiotic glucuronidation | 5 | 4.06E-08 | UGT1A1/UGT1A10/UGT1A3/UGT1A4/UGT1A5 |
|  | uronic acid metabolic process | 6 | 1.04E-07 | SORD/UGT1A1/UGT1A10/UGT1A3/UGT1A4/UGT1A5 |
|  | glucuronate metabolic process | 6 | 1.04E-07 | SORD/UGT1A1/UGT1A10/UGT1A3/UGT1A4/UGT1A5 |
|  | flavonoid metabolic process | 5 | 2.56E-07 | UGT1A1/UGT1A10/UGT1A3/UGT1A4/UGT1A5 |
|  | cellular glucuronidation | 5 | 9.59E-07 | UGT1A1/UGT1A10/UGT1A3/UGT1A4/UGT1A5 |
|  | morphogenesis of embryonic epithelium | 9 | 1.85E-05 | ALX1/CTHRC1/GRHL2/LHX2/OVOL2/PCDH8/SIX4/SOX11/TFAP2A |
|  | response to xenobiotic stimulus | 12 | 2.66E-05 | ABAT/EPO/PCK1/SLC18A2/SLITRK5/TAC1/TDO2/UGT1A1/UGT1A10/UGT1A3/UGT1A4/UGT1A5 |
|  | extracellular structure organization | 14 | 5.20E-05 | CD36/COL23A1/COL4A4/COL9A2/CPB2/GPM6B/HAPLN1/HPSE2/LAMA4/LPA/NDNF/NPHS1/PDGFRA/TGFBI |
|  | action potential | 8 | 5.61E-05 | ATP1A1/CAV1/GJC1/NTRK2/SCN1A/SCN2B/SCN7A/TAC1 |
|  | morphogenesis of an epithelium | 15 | 9.29E-05 | ALX1/CTHRC1/DLL4/FGF1/GRHL2/HOXD13/LHX2/NKD1/OVOL2/PCDH8/PGF/SIX4/SOX11/TFAP2A/ZIC3 |
| GO-CC | cell-cell junction | 15 | 3.08E-05 | ATP1A1/CDH9/CGN/CLDN16/CLDN8/GJC1/GRHL2/IGSF5/ITK/KIAA1210/LCP2/NPHS1/OCLN/PCDH9/SCN1A |
|  | collagen trimer | 6 | 0.0002 | COL23A1/COL4A4/COL9A2/CTHRC1/GLDN/MSR1 |
|  | voltage-gated sodium channel complex | 3 | 0.0003 | SCN1A/SCN2B/SCN7A |
|  | extracellular matrix | 14 | 0.0003 | ADAMTS10/COL23A1/COL4A4/COL9A2/CTHRC1/FGF1/HAPLN1/HPSE2/LAMA4/LRRTM4/NDNF/PCSK6/TGFBI/WISP2 |
| GO-MF^*^ | sodium ion transmembrane transporter activity | 10 | 3.43E-06 | ATP1A1/SCN1A/SCN2B/SCN7A/SLC12A3/SLC17A2/SLC18A2/SLC1A3/SLC5A7/SLC9A2 |
|  | transcriptional activator activity, RNA polymerase II transcription regulatory region sequence-specific binding | 16 | 1.64E-05 | ALX1/BARX2/DLX3/DLX5/DMRT1/GRHL2/HOXD13/LHX2/OVOL2/RFX6/SIX4/SOX11/SPI1/TFAP2A/TFAP2C/ZIC3 |
|  | glucuronosyltransferase activity | 5 | 1.73E-05 | UGT1A1/UGT1A10/UGT1A3/UGT1A4/UGT1A5 |
|  | secondary active transmembrane transporter activity | 10 | 0.0001 | SLC12A3/SLC16A1/SLC16A3/SLC17A2/SLC18A2/SLC1A3/SLC26A4/SLC2A12/SLC5A7/SLC9A2 |
|  | symporter activity | 8 | 0.0001 | SLC12A3/SLC16A1/SLC16A3/SLC17A2/SLC18A2/SLC1A3/SLC2A12/SLC5A7 |
|  | metal ion transmembrane transporter activity | 14 | 0.0002 | ATP1A1/CLDN16/KCNK10/SCN1A/SCN2B/SCN7A/SLC12A3/SLC17A2/SLC18A2/SLC1A3/SLC30A10/SLC5A7/SLC9A2/TRPM8 |
|  | monovalent inorganic cation transmembrane transporter activity | 12 | 0.0007 | ATP1A1/KCNK10/SCN1A/SCN2B/SCN7A/SLC12A3/SLC17A2/SLC18A2/SLC1A3/SLC2A12/SLC5A7/SLC9A2 |
|  | solute:cation symporter activity | 6 | 0.0007 | SLC12A3/SLC17A2/SLC18A2/SLC1A3/SLC2A12/SLC5A7 |
|  | carboxylic acid binding | 8 | 0.00087 | HAPLN1/PCK1/PMP2/SLC1A3/ST8SIA4/TDO2/UGT1A1/UGT1A3 |
|  | solute:sodium symporter activity | 5 | 0.0009 | SLC12A3/SLC17A2/SLC18A2/SLC1A3/SLC5A7 |
| KEGG | Pentose and glucuronate interconversions | 6 | 4.37E-05 | UGT1A10, SORD, UGT1A3, UGT1A5, UGT1A4, UGT1A1 |
|  | Ascorbate and aldarate metabolism | 5 | 2.93E-04 | UGT1A10, UGT1A3, UGT1A5, UGT1A4, UGT1A1 |
|  | Retinol metabolism | 6 | 0.0010 | RDH12, UGT1A10, UGT1A3, UGT1A5, UGT1A4, UGT1A1 |
|  | Porphyrin and chlorophyll metabolism | 5 | 0.0016 | UGT1A10, UGT1A3, UGT1A5, UGT1A4, UGT1A1 |
|  | Drug metabolism - other enzymes | 5 | 0.0023 | UGT1A10, UGT1A3, UGT1A5, UGT1A4, UGT1A1 |
|  | Steroid hormone biosynthesis | 5 | 0.0053 | UGT1A10, UGT1A3, UGT1A5, UGT1A4, UGT1A1 |
|  | Drug metabolism - cytochrome P450 | 5 | 0.0093 | UGT1A10, UGT1A3, UGT1A5, UGT1A4, UGT1A1 |
|  | Metabolism of xenobiotics by cytochrome P450 | 5 | 0.0125 | UGT1A10, UGT1A3, UGT1A5, UGT1A4, UGT1A1 |
|  | Chemical carcinogenesis | 5 | 0.0162 | UGT1A10, UGT1A3, UGT1A5, UGT1A4, UGT1A1 |
|  | Tight junction | 5 | 0.0214 | CLDN16, CLDN8, IGSF5, OCLN, CGN |

The GO and KEGG analysis of mRNAs in ceRNA network were carried out using an online tool, Database for Annotation, Visualization and Integrated Discovery. The results showed that these mRNAs were significantly enriched in 47 GO-BP terms, 4 GO-CC terms, 25 GO-MF terms and 12 KEGG pathways. ^*^ represents the top 10 GO-BP/GO-MF terms and KEGG pathways. ^*^ indicated that the *P* value was adjusted by Benjamini & Hochberg method.

GO, Gene Ontology; BP, biological process; CC, cellular component; MF, molecular function; KEGG: Kyoto Encyclopedia of Genes and Genomes; ceRNA, competing endogenous RNA.

**Table S4 The relationships of miRNA-21/miRNA-155 and corresponding lncRNAs.**

| MiRNA | Symbol | Correlation | *P* value | MiRNA | Symbol | Correlation | *P* value |
| --- | --- | --- | --- | --- | --- | --- | --- |
| hsa-mir-21 | LINC00472 | -0.337 | 5.57E-15 | hsa-mir-155 | SLC25A5.AS1 | -0.212 | 1.34E-06 |
| hsa-mir-21 | SLC25A5.AS1 | -0.331 | 1.77E-14 | hsa-mir-155 | LINC00472 | -0.193 | 1.13E-05 |
| hsa-mir-21 | TCL6 | -0.284 | 6.28E-11 | hsa-mir-155 | TCL6 | -0.154 | 0.000 |
| hsa-mir-21 | LINC00443 | -0.228 | 1.82E-07 | hsa-mir-155 | LINC00443 | -0.134 | 0.002 |
| hsa-mir-21 | COL18A1.AS1 | -0.221 | 4.77E-07 | hsa-mir-155 | FGF12.AS2 | -0.132 | 0.003 |
| hsa-mir-21 | LINC00507 | -0.188 | 1.91E-05 | hsa-mir-155 | AP005717.1 | -0.110 | 0.013 |
| hsa-mir-21 | C12orf77 | -0.141 | 0.001 | hsa-mir-155 | LINC00461 | -0.105 | 0.019 |
| hsa-mir-21 | LINC00461 | -0.122 | 0.006 | hsa-mir-155 | LINC00507 | -0.101 | 0.023 |
| hsa-mir-21 | AP005717.1 | -0.105 | 0.017 | hsa-mir-155 | COL18A1.AS1 | -0.063 | 0.157 |
| hsa-mir-21 | FGF12.AS2 | -0.094 | 0.034 | hsa-mir-155 | C12orf77 | -0.050 | 0.259 |
| hsa-mir-21 | WT1.AS | -0.071 | 0.109 | hsa-mir-155 | UCA1 | -0.044 | 0.319 |
| hsa-mir-21 | LATS2.AS1 | -0.059 | 0.181 | hsa-mir-155 | WT1.AS | 0.028 | 0.526 |
| hsa-mir-21 | GLIS3.AS1 | -0.010 | 0.824 | hsa-mir-155 | TRIM36.IT1 | 0.033 | 0.461 |
| hsa-mir-21 | TRIM36.IT1 | -0.006 | 0.885 | hsa-mir-155 | HOTTIP | 0.055 | 0.219 |
| hsa-mir-21 | BPESC1 | 0.004 | 0.926 | hsa-mir-155 | BPESC1 | 0.057 | 0.203 |
| hsa-mir-21 | AC105206.1 | 0.046 | 0.297 | hsa-mir-155 | AC020907.1 | 0.058 | 0.190 |
| hsa-mir-21 | AC004832.1 | 0.053 | 0.228 | hsa-mir-155 | AC004832.1 | 0.089 | 0.045 |
| hsa-mir-21 | HOTTIP | 0.099 | 0.025 | hsa-mir-155 | C20orf203 | 0.090 | 0.043 |
| hsa-mir-21 | UCA1 | 0.105 | 0.018 | hsa-mir-155 | NALCN.AS1 | 0.106 | 0.017 |
| hsa-mir-21 | NALCN.AS1 | 0.120 | 0.007 | hsa-mir-155 | AC009093.1 | 0.119 | 0.007 |
| hsa-mir-21 | AC009093.1 | 0.144 | 0.001 | hsa-mir-155 | AL356356.1 | 0.145 | 0.001 |
| hsa-mir-21 | MIAT | 0.154 | 0.000 | hsa-mir-155 | LINC00313 | 0.153 | 0.001 |
| hsa-mir-21 | C20orf203 | 0.157 | 0.000 | hsa-mir-155 | LINC00475 | 0.166 | 0.000 |
| hsa-mir-21 | AL356356.1 | 0.168 | 0.000 | hsa-mir-155 | LATS2.AS1 | 0.173 | 8.82E-05 |
| hsa-mir-21 | AC020907.1 | 0.179 | 4.67E-05 | hsa-mir-155 | GLIS3.AS1 | 0.177 | 5.60E-05 |
| hsa-mir-21 | LINC00299 | 0.201 | 4.96E-06 | hsa-mir-155 | AC105206.1 | 0.183 | 3.35E-05 |
| hsa-mir-21 | AC105020.1 | 0.211 | 1.51E-06 | hsa-mir-155 | LINC00299 | 0.188 | 1.92E-05 |
| hsa-mir-21 | VCAN.AS1 | 0.247 | 1.58E-08 | hsa-mir-155 | VCAN.AS1 | 0.194 | 1.04E-05 |
| hsa-mir-21 | MIR155HG | 0.262 | 2.01E-09 | hsa-mir-155 | PVT1 | 0.200 | 5.50E-06 |
| hsa-mir-21 | LINC00313 | 0.276 | 2.27E-10 | hsa-mir-155 | AC105020.1 | 0.202 | 4.36E-06 |
| hsa-mir-21 | AC016773.1 | 0.283 | 8.20E-11 | hsa-mir-155 | MIAT | 0.220 | 5.52E-07 |
| hsa-mir-21 | FAM13A.AS1 | 0.285 | 5.22E-11 | hsa-mir-155 | FAM13A.AS1 | 0.223 | 3.76E-07 |
| hsa-mir-21 | PVT1 | 0.347 | 7.49E-16 | hsa-mir-155 | SNHG12 | 0.307 | 1.38E-12 |
| hsa-mir-21 | hsa-mir-155 | 0.349 | 4.92E-16 | hsa-mir-155 | AC016773.1 | 0.316 | 3.00E-13 |
| hsa-mir-21 | LINC00475 | 0.367 | 9.87E-18 | hsa-mir-155 | hsa-mir-21 | 0.349 | 4.92E-16 |
| hsa-mir-21 | SNHG12 | 0.424 | 1.28E-23 | hsa-mir-155 | MIR155HG | 0.754 | 1.14E-94 |
| hsa-mir-21 | hsa-mir-21 | 1 | 0 | hsa-mir-155 | hsa-mir-155 | 1 | 0 |

miRNA,microRNA; lncRNAs, long non-coding RNAs.
